# Hepatitis C Transmission after Prostate Biopsy

**DOI:** 10.1155/2013/797248

**Published:** 2013-02-21

**Authors:** Karim Ferhi, Morgan Rouprêt, Pierre Mozer, Guillaume Ploussard, Alain Haertig, Alexandre de La Taille

**Affiliations:** ^1^Academic Department of Urology, Pitie-Salpetriere Hospital, University Paris VI, Boulevard de l'Hopital, 75013 Paris, France; ^2^Academic Department of Urology, Henri Mondor Hospital, Paris Est Creteil University, Avenue de Lattre de Tassigny, 94010 Creteil, France

## Abstract

Prostate biopsy is a current and well-codified procedure; antibiotic prophylaxis and rectal enema limit the risk of infection. To date, there has been no reported viral transmission between patients due to a contaminated ultrasound probe. In this study, we report the case of a patient who contracted the hepatitis C virus after transrectal prostate biopsy as part of an individual screening for prostate cancer.

## 1. Introduction

In urology, prostate biopsy is currently the only means to confirm a diagnosis of prostate cancer, the most prevalent cancer found in men. Approximately from 120,000 to 150,000 biopsies are conducted each year in France, resulting in 50,000 new diagnoses of prostate cancer. Prostate biopsies are primarily conducted transrectally, and the risk of infection with an antibiotic prophylaxis such as quinolone is estimated to be between 2 and 5% (versus 30 to 40% without an antibiotic prophylaxis) [1]. This risk of infection is linked to the procedure itself, which is conducted in a septic environment. To date, there has been no reported risk of viral transmission between patients due to contaminated ultrasound probes. Although this subject has not been systematically studied, the risk of infection in these procedures can be considered very low. In this study, we report the case of a patient who contracted the hepatitis C virus after a transrectal prostate biopsy as part of an individual screening for prostate cancer.

## 2. Clinical Case

Mr. S., 53 years of age without any particular medical history, received an ultrasound-guided prostate biopsy as part of an individual screening for prostate cancer. He had a PSA of 5.62 ng/mL and a nonsuspicious digital rectal examination (prostate estimated at 60 g). The biopsy was conducted without any special procedures such as sterile ECBU or urine culture. The anatomopathological report did not show any outbreak of carcinomatous proliferation. Four weeks after the biopsy, the patient presented a pseudo-influenza syndrome associated with hepatic cytolysis (20 times normal). The etiological workup showed discretely positive anti-HCV antibodies and a C virus PCR of 6 log. Other viral serologies, such as A, B, and mononucleosis, were negative (the last transaminases performed in this patient in 2007 were normal). The diagnosis of acute hepatitis C was then raised. The etiological investigation of Mr. S. was noted by the absence of transfusions and intravenous (IV) drug abuse as well as various risk factors usually associated with this condition. Moreover, the results of serological tests performed on patients who received prostate biopsies in the three days preceding the biopsy of Mr. S. all came back negative, as did those of the sampling doctor and the consulting nurse. The internal report by the CLIN of the establishment concerned revealed that nearly 36% of the protective sheaths were perforated after biopsies and that nondisposable, reusable metal puncture guides that could have been contaminated were used. The report of the forensic expert stated (on the advice of virology experts) that during the cleaning of medical devices, especially endoscopes and ultrasound probes, an organic film deposited on the apparatus during sterilization can protect a virus for a relatively long time, between 10 and 15 days at the most, and that it is therefore not impossible that the contamination of Mr. S. could have occurred via a probe or guide that was used up to two weeks prior to the biopsy in question.

Faced with this array of arguments, the establishment believes that Mr. S. was likely contaminated during his prostate biopsy and will therefore be compensated accordingly. 

## 3. Discussion 

The nosocomial and nontransfusional transmission of HCV has become an important means of infection, though it remains secondary to contamination by IV drug abuse (70% of the 50,000 new cases annually) [2]. 

The risk of nosocomial transmission of HCV seems to be limited to invasive medicosurgical procedures *using nondisposable, poorly sterilized equipments*.** A national survey conducted in 1994 in the hospital environment suggested a possible nosocomial, nontransfusional infection in 15% of the hepatitis C cases studied, which occurred more often in women than in men (19% versus 12%) [3]. Medicosurgical procedures after which transmission of HCV has been reported in the literature include digestive and possibly bronchial endoscopy (especially if accompanied by biopsies), interventional radiology (e.g., retrograde cholangiography), transurethral prostatic resections, and some anesthesia. In addition, dental care, medically assisted procreation (MAP), acupuncture, mesotherapy, and all procedures that may place the blood of an infected subject in contact with that of a noninfected subject must also be suspected, even if transmission has not been demonstrated [4]. 

Cases of transmission of HCV by endorectal prostate biopsy are extremely rare. *There are currently no cases of such transmission in the literature*. Only one prospective study, which was conducted recently, has evaluated the risks of viral transmission (hepatitis B and C and HIV) during prostate biopsies. None of the 528 patients who were biopsied during this study between January 2003 and January 2006 showed seroconversion to hepatitis C [5]. 

Experts have noted the existence of a “bio film” that could protect and trap the virus on the protection guide used in biopsies and other procedures. In fact, during the investigation of a patient infected with HCV, stains consisting of blood, secretions, and remains of mucosa after biopsy were deposited even in the inner channel of the guide. Even if the instruments are cleaned immediately after the procedure, manual washing does not always eliminate organic contaminants. It is possible that oxidizing disinfectants, which act on the surface of debris, form a “bio film,” a protective covering that prevents the thorough action of the disinfectants. During subsequent use, the debris and its bio film could detach and contaminate the next patient. This mode of contamination is more common when the washing time is short, when the delay between contamination and washing is long, and when the equipment is old or scratched. Currently, the only way to completely avoid transmission linked to a “bio film” is to use new equipment for every examination, which is not practical for certain procedures such as endoscopies and laryngoscopies. This contamination is therefore theoretically more common when the hospital service accommodates carrier patients [6]. 

Experts have also revealed that nearly one-third of protective sheaths are perforated. Use of these protective shields was evaluated by the High Council of Public Health in 2007. This study initially specified that bacterial and viral transmissions in particular were extremely rare during tests such as prostate biopsies. The only reported case of infection was of a bacterial transmission of *Pseudomonas aeruginosa*. The investigation showed that the origin of the infection was linked to contamination of the biopsy needle guide, which, after disinfection, was rinsed under running water and not cleaned with a brush. This paper recommends that the guide and needle be considered critical medical disposables. The needle must be used only once, and the guide must be sterilized or otherwise undergo high-level disinfection followed by rinsing with sterile water [7]. 

The integrity of protective sheaths, measured by the rate of perforation, has been the subject of several studies, particularly concerning transvaginal and transrectal probes. These studies, which include comparative and noncomparative and randomized and nonrandomized studies, focused on protective sheaths or condoms (used as sheaths). Perforation is detected either visually or by tests, such as leakage of water or of hydrogen peroxide, that differ according to the study. Overall rates of perforation range from 1 to 8% and vary according to the type of protection tested and the detection method [8–10]. However, none of these studies evaluated the infectious risk of contamination. 

Another point to note is that microorganisms can also be preserved and protected on biopsy guides for several days by the “bio film.” International recommendations call for prostate biopsies using a single-use biopsy guide or, if it is reusable, to sterilize it in an autoclave with water vapor. In 2006, an investigation conducted by several hospital services alerted the American authorities, particularly the FDA, to numerous failures in sterilization of medical equipment used in prostate biopsies [11]. 

The FDA [12] restated the necessity of adhering to sterilization regulations and insisted on the importance of thoroughly sterilizing reusable puncture guides and using the specified brushes supplied with these guides. These brushes allow proper cleaning of these guides, particularly of the light, which can harbor pathogens. The FDA stipulates, among other things, that it is necessary to sterilize and clean each part of the guide separately and to verify visually that no residue remains. Use of sterile water is preferred for rinsing the channels at the end of the procedure. Some authors [13] have shown that the use of single-use puncture guides can decrease the risk of bacterial contamination. They compared two groups of patients who had undergone ultrasound prostate biopsies, one with single-use guides and the other with reusable guides. The first group had lower rates of asymptomatic bacterial infection, fever, and urinary infections than the second group (4.5% versus 9%, 5% versus 10%, and 2% versus 9%, resp.). 

## 4. Conclusion

This reported case of contamination following a prostate biopsy should encourage practitioners to scrupulously adhere to the recommendations regarding existing sterilization norms, to use protective sheaths, to verify the integrity of protective sheaths after use, and to use single-use puncture guides.
